# Safety and Feasibility of Rotational Atherectomy for Retrograde Recanalization of Chronically Occluded Coronary Arteries

**DOI:** 10.3389/fcvm.2022.854757

**Published:** 2022-06-17

**Authors:** Jing Wang, Junlin Huang, Abdul-Subulr Yakubu, Kaize Wu, Zehan Huang, Zhian Zhong, Hongtao Liao, Bin Zhang

**Affiliations:** ^1^School of Medicine, South China University of Technology, Guangzhou, China; ^2^Department of Cardiology, Guangdong Cardiovascular Institute, Guangdong Provincial People's Hospital, Guangdong Academy of Medical Sciences, Guangzhou, China; ^3^Tamale Teaching Hospital, Tamale, Ghana; ^4^Department of Cardiovascular Medicine, Affiliated Nanhai Hospital, Southern Medical University (People's Hospital of Nanhai District), Foshan, China; ^5^Department of Cardiovascular Medicine, The Second Affiliated Hospital of Guangzhou Medical University, Guangzhou, China

**Keywords:** chronic total occlusion, percutaneous coronary intervention, in-hospital outcomes, long-term outcomes, retrograde, rotational atherectomy

## Abstract

**Objective:**

To evaluate the safety and feasibility of rotational atherectomy (RA) in retrograde chronic total occlusion percutaneous coronary intervention (CTO-PCI) by analyzing immediate and long-term outcomes.

**Background:**

Recent evidence supports the safety and feasibility of RA in CTO-PCI. However, few studies have focused on the use of RA in a retrograde approach to percutaneous revascularization of chronic total occlusion (CTO) lesions and information on long-term outcomes is lacking.

**Methods:**

A total of 329 patients who underwent retrograde CTO-PCI, out of 1496 consecutive CTO-PCI patients from April 2017 to July 2020, were retrospectively recruited from the 2nd Cardiology Department of the Guangdong Provincial People's Hospital. 16 patients underwent RA (RA group) whilst 313 did not (non-RA group).

**Results:**

Technical (87.5% vs. 87.5) and procedural (85.9% vs. 87.5) success rates were similar between both groups. There was no difference concerning major procedural complications between groups (12.5% vs. 19.2%; *p* > 0.75). No in-hospital MACCEs was recorded in the RA group while there were eight MACCEs in the non-RA group (*p* > 0.99). In the RA group, 2 cases recorded perforation (1 target vessel perforation case and 1 branch vessel perforation), and 55 cases of vessel perforations/dissections were recorded in non-RA group including 18 target vessel perforations, 2 branch vessel perforations, 35 collateral vessel perforations (one patient died from cardiac tamponade). No difference was found in terms of the perforation rate between the two groups (*p* > 0.99). Over a mean follow-up period of 26.47 ± 14.46 months, use of RA in retrograde CTO-PCI did not result in an increased mortality rate [hazard ratio (HR) 1.58, 95% confidence interval (CI), 0.31–8.21, *p* = 0.65], major adverse cardiac and cerebral events (HR 0.99, 95% CI 0.35–2.79, *p* = *0*.99) or overall rehospitalization rate (HR 1.27, 95% CI 0.44–3.67, *p* = 0.67). Adjusted Kaplan–Meier curves according to Cox regression model suggested several predictors influencing the all-cause mortality, cardiovascular mortality, MACCEs, stroke rate, non-fatal myocardial infarction, target vessel recanalization rate and rehospitalization rate in the comparison.

**Conclusions:**

Our study demonstrates that the in-hospital outcomes and long-term follow up events were the same between RA and non-RA retrograde CTO-PCI patients. RA offered an option for skillful operators in difficult cases when the lesion was severely calcified in retrograde CTO-PCI.

## Introduction

With advancements in technique and equipment, the success rate of chronic total occlusion percutaneous coronary intervention (CTO-PCI) has greatly improved over the years (1). Despite this, severely calcified coronary artery lesions remain a common cause of failure of equipment delivery and balloon expansion during chronic total occlusion (CTO) recanalization (2–6). Evidence of the viability and safety of CTO-PCI for calcified lesions using the antegrade approach abounds (7–9). Reverse controlled antegrade and retrograde subintimal tracking (reverse CART) is the most common retrograde CTO crossing technique in most contemporary series (66% in a multicenter U.S. registry) (10). The use of retrograde crossing techniques, particularly reverse CART, in severely calcified lesions during retrograde CTO-PCI has been considered to confer a relatively high risk of dissection and perforation following subsequent rotational atherectomy (RA) in these lesions. Azzalini et al. proposed the concept of vessel architecture, which sought to distinguish coronary structures (occlusive plaque and adventitia) from the extravascular space, and suggested that CTO-PCI can be carried out safely and effectively as long as one remains within the subadventitial space (11). The feasibility of RA in the subadventitial space during CTO-PCI has been suggested (12, 13). The present study sought to further evaluate the safety and feasibility of RA during CTO-PCI using the retrograde approach.

## Methods

### Study Population

**Figure 1:**
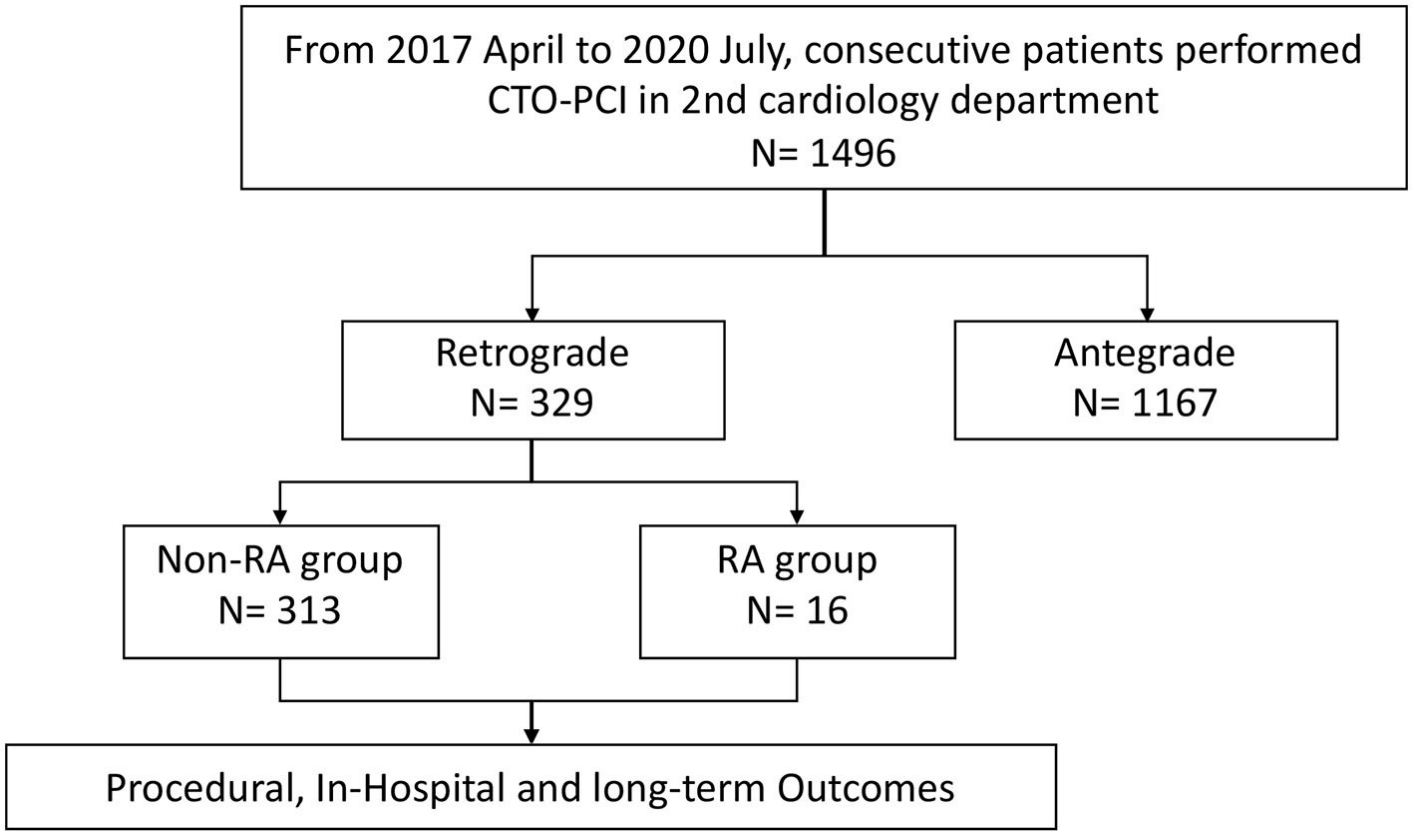
Flow chart of the study population.

**Figure 2:**
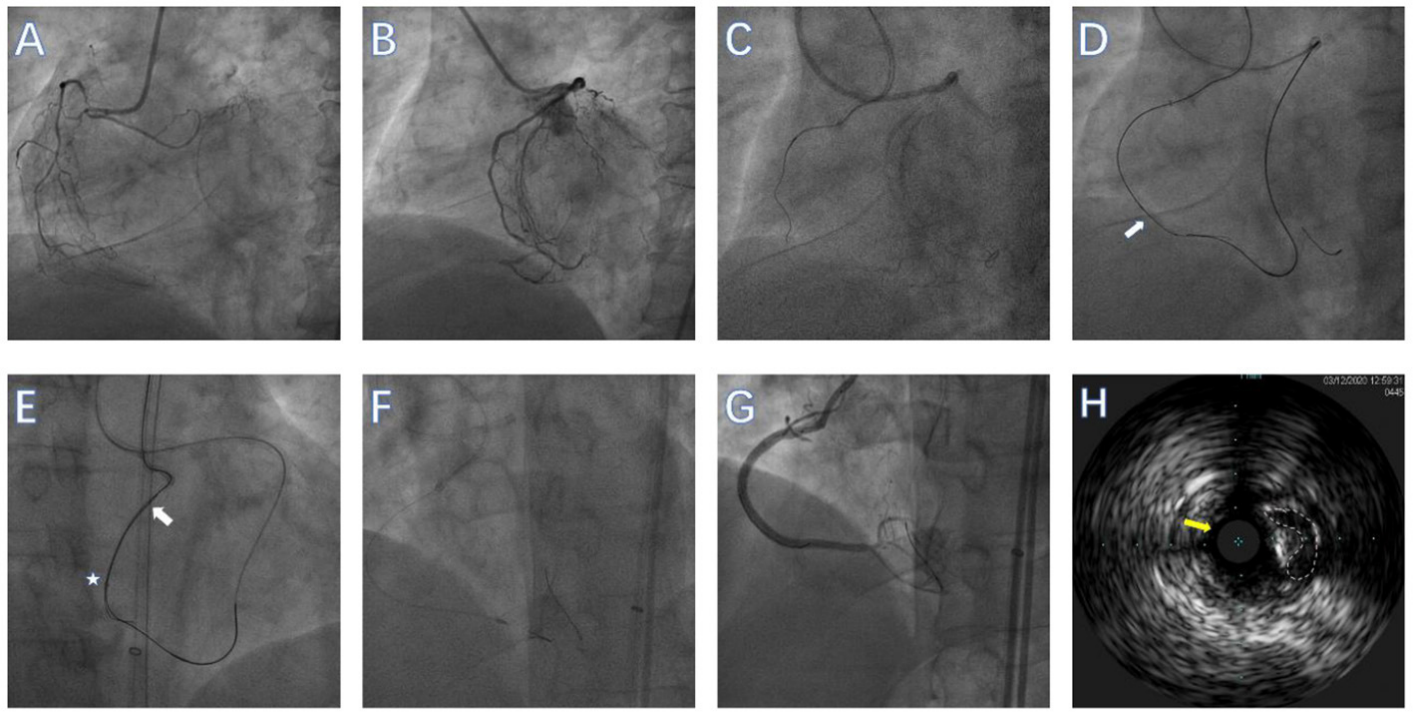
Performing Guidezilla^TM^ reverse controlled antegrade and retrograde subintimal tracking (CART) during rotational atherectomy (RA) in a right coronary artery (RCA) CTO lesion. **(A)** RCA in left anterior oblique view showing an ambiguous proximal cap without obvious calcification. **(B)** RCA in left anterior oblique view showing the distal cap of the RCA. **(C)** Antegrade wire in the subadventitial space of mid-RCA. **(D)** The CTO lesion was crossed over using the Guidezilla^TM^ reverse CART technique (the white arrow represents the dilated balloon). **(E)** Retrograde wire (white arrow) was advanced into the Guidezilla^TM^ (white star) of the RCA and externalized. **(F)** Rotational atherectomy was then performed using a 1.25-mm burr. **(G)** Angiography after successful CTO-PCI. **(H)** Intravascular ultrasound confirmed that the guidewire (yellow arrow) was in the subadventitial space (The white dotted line represents the true lumen of the vessel).

In this single-center, retrospective, cohort study, the records of patients who underwent CTO-PCI using the retrograde approach from April 2017 to July 2020 in the 2nd Cardiology Department of Guangdong Provincial People's Hospital were reviewed. The operators performed more than 200 CTO cases per year and with a success rate of about 90%. CTO-PCI was performed on 1496 consecutive patients, of which 329 patients matched the eligibility criteria and were included in our study. RA was used in 16 of the 329 patients because of failure of equipment crossing or balloon undilation in severely calcified stenotic lesions. Figure 1 shows the flow chart of the study population. The eligibility criteria for the study were: (1) age of 18 years or older; (2) an indication for CTO-PCI, including angina symptoms and/or evidence of reversible myocardial ischemia by perfusion imaging or stress testing; and (3) All cases had failed antegrade wire escalation. Patients were excluded if they were older than 85 years or were not the suitable candidates because of severe hemorrhagic disease or intolerance to dual antiplatelet therapy. Demographic, angiographic, procedural, and in-hospital data were obtained from the catheterization laboratory database and hospital charts. Figure 2 demonstrates a case of retrograde CTO-PCI using RA.

### Definitions

A coronary CTO was defined as total occlusion of a coronary artery segment with Thrombolysis in Myocardial Infarction (TIMI) flow grade 0 and an estimated duration of more than 3 months, with angiographic evidence. The duration of occlusion was estimated according to the onset of clinical symptoms or previous myocardial infarction (MI) with angiographic evidence. The Japanese-CTO (J-CTO) score and PROGRESS-CTO score were used to assess CTO lesions. Werner classification was used to assess collateralization (14–16). Technical success of retrograde CTO-PCI was defined as residual stenosis <30% and antegrade TIMI flow grade 3. Procedural success was regarded as technical success with no in-hospital major adverse cardiac and cerebral events (MACCEs). In-hospital events were defined as death, periprocedural MI, urgent target vessel revascularization (including repeat PCI or coronary artery bypass graft), pericardiocentesis, cardiac tamponade requiring surgery and stroke. During follow-up, MACCE was defined as cardiovascular death, non-fatal MI, ischemia-driven target vessel recanalization and stroke. The criteria for MI were based on the new Fourth Universal Definition of MI (17). Stent thrombosis was defined in accordance with the Academic Research Council criteria (18).

### Interventional Procedures

Before and after CTO-PCI, all patients received optimal dual antiplatelet therapy (aspirin 100 mg once daily and clopidogrel 75 mg once daily or ticagrelor 90 mg twice daily). Patients received an initial bolus of intravenous unfractionated heparin (150 IU/kg) during the procedure; additional boluses were given to maintain an activated clotting time (ACT) >300 s, which was monitored every 30 min. At the operator's discretion, additional doses of a glycoprotein IIb/IIIa (GPIIb/IIIa) inhibitor were administered selectively. The choice of vascular access depended on the operator's personal preference as well as anatomical considerations. Retrograde recanalization techniques and equipment were used at the operator's discretion. RA was used following failure of balloon crossing or expansion, or after balloon rupture or failure of other equipment to cross after wire externalization. Rota wire® (Boston Scientific Corp) was exchanged after the extraction of wires *via* a Finecross® microcatheter (Terumo Company, Japan) or Corsair® (Asahi Intec, Japan) microcatheter. If the microcatheter failed to cross, Rota wire® was manipulated to primarily cross the CTO lesion (19). Retrograde angiography was performed to ensure that the Rota wire was in the true lumen. The size of the burr used was at the discretion of the operator. The rotational speed of RA was between 160000 and 200000 rotations per minute (RPM). Balloon pre-dilatation was performed after successful RA, followed by drug eluting stent implantation.

### Follow-Up

Follow-up data was collected by telephonic interviews years after PCI as well as through the revision of clinical documentation when patients returned for further consultation. All data collection and use of patient data were done in accordance with the Declaration of Helsinki and approved by the Research Ethics Committee of Guangdong Provincial people's hospital and Guangdong Academy of Medical Sciences [No. GDREC2017196H(R1)] in July 2017.

### Statistical Analysis

Quantitative data are reported as means ± SD and tested by the Student *t*-test. Pearson χ2 test or Fisher's exact test was used to analyze differences in qualitative data for discrete variables. The Kaplan–Meier method was used to calculate and graphically describe the free rates of MACCE, MI, stroke, survival, and rehospitalization of the two groups. The multivariable Cox regression analysis model was built by stepwise selection. All baseline and procedural patient variables in univariable analysis defined by *p* < 0.1 were entered into the stepwise model. Differences with a *p* < 0.05 were regarded as statistically significant. All statistical analyses were performed using the SPSS software package, version 20.0 (SPSS Inc, Chicago, IL) and GraphPad Prism version 9 (GraphPad Software Inc., San Diego, CA, USA).

## Results

### Baseline Clinical Characteristics

A total of 329 patients who fulfilled the inclusion criteria were included in the study. Clinical characteristics of the study population are shown in Table 1. Sixteen patients underwent RA during retrograde CTO-PCI while 313 patients had retrograde CTO-PCI without RA. The distribution of clinical characteristics between the two groups was not different. The mean age was 59.70 ± 10.52 years in the non-RA group and 60.87 ± 9.82 in the RA group, and more than 90% were male in both groups. There were no differences in the prevalence of hypertension, hyperlipidemia, smoking, diabetes mellitus or history of prior PCI or coronary artery bypass graft between the two groups. Renal function and ejection fraction did not differ significantly between the two groups.

**Table 1:** Baseline clinical characteristics.

**Variable**	**Non-RA** **(*n* = 313)**	**RA** **(*n* = 16)**	*P*-value^*^
Age (years)	59.70 ± 10.52	60.87 ± 9.82	0.43
Male, *n* (%)	284 (90.7)	16 (100)	0.38
Diabetes mellitus, *n* (%)	95 (30.4)	8 (50)	0.10
Dyslipidemia, *n* (%)	89 (28.4)	5 (31.3)	0.81
Hypertension, *n* (%)	184 (58.8)	14 (87.5)	0.03
Current smoker, *n* (%)	70 (22.4)	3 (18.8)	>0.99
Prior MI, *n* (%)	79 (25.2)	4 (25)	>0.99
Prior PCI, *n* (%)	206 (65.8)	10 (62.5)	0.79
Prior CABG, *n* (%)	15 (4.8)	0 (0)	>0.99
LVEF (%)	54.34 ± 12.73	52.44 ± 11.50	0.38
LVEF <50%, *n* (%)	90 (28.8)	5 (31.3)	0.83
Serum creatinine, μmol/L	101.32 ± 89.76	98.14 ± 33.51	0.76

*MI, myocardial infarction; PCI, percutaneous coronary artery intervention; CABG, coronary artery bypass surgery; LVEF, left ventricular ejection fraction.*

* *p < 0.05 is considered significant*.

### Angiographic Characteristics

The angiographic characteristics of the patients' coronary lesions are described in Table 2. Compared to the non-RA group, RA patients had higher prevalence of moderate/severe calcifications (100% in RA vs. 50.2% in non-RA; *p* < 0.0001) and moderate/severe tortuosity (87.5% vs. 58.8%; *p* = 0.03) at the CTO lesions. There was no difference between the RA and non-RA groups with regards to the distribution of the CTO target vessel. Though most of the CTO lesions requiring retrograde PCI were in the right coronary artery, it did not suggest significant difference (75% and 59.1%, *p* = 0.3). J-CTO score (3.88 ± 0.89 vs. 2.84 ± 1.03, *p* = 0.25) and PROGRESS CTO score (2.19 ± 0.66 vs. 2.01 ± 0.81, *p* = 0.67) did not differ between the two groups, yet the scores were higher in the RA group. No difference was found in Werner score (0.81 ± 0.75 vs. 1.41 ± 0.67, *p* = 0.95) between the two groups.

**Table 2:** Angiographic characteristics.

**Variable**	**Non-RA** **(*n* = 313)**	**RA** **(*n* = 16)**	*P*-value^*^
Target-vessel CTO			
LAD, *n* (%)	120 (38.3)	4 (25)	0.43
LCX, *n* (%)	7 (2.2)	0 (0)	>0.99
RCA, *n* (%)	185 (59.1)	12 (75)	0.30
LM, *n* (%)	1 (0.3)	0 (0)	>0.99
Multivessel, *n* (%)	263 (84)	16 (100)	0.14
Multiple CTO, *n* (%)	72 (23)	7 (43.8)	0.06
Blunt stump, *n* (%)	221 (70.6)	12 (75)	>0.99
Moderate/severe tortuosity	184 (58.8)	14 (87.5)	0.03 <0.05
Moderate/severe calcification	157 (50.2)	16 (100)	<0.0001
Lesion length >20 mm	276 (88.2)	16 (100)	0.23
Prior failed CTO PCI, *n* (%)	97 (31)	4 (25)	0.78
J-CTO score	2.84 ± 1.03	3.88 ± 0.89	0.25
Progress CTO score	2.01 ± 0.81	2.19 ± 0.66	0.67
Werner score	1.41 ± 0.67	0.81 ± 0.75	0.95

*CTO, Chronic Total Occlusion; LAD, Left Anterior Descending; LCX, Left Circumflex; RCA, Right Coronary Artery; LM, Left Main.*

* *p < 0.05 is considered significant*.

### Procedural Characteristics

Table 3 showed the procedural characteristics of the two groups. There was a higher trend toward use of reverse CART in successful crossing strategy (75% in RA vs. 59.4% in non-RA; *p* = 0.29), and retrograde wire escalation tended to be lower in RA patients (18.8% vs. 27.2%; *p* = 0.57). In the RA group guide catheter extension (Guidezilla^TM^, Boston Scientific, Natick, USA) was more frequently applied, as compared with non-RA subjects (75% vs. 46%, *p* = 0.04). IVUS use was not different in cases of both cohorts. Septal collateral channel was the common interventional collateral channel in both retrograde PCI groups and showed no significant difference. Epicardial collateral channel tended to be applied more often in non-RA group (18.8% vs. 25.6%; *p* = 0.77). There was no significant difference in the number and length of stents implanted between the two groups. The main indications for RA during retrograde CTO-PCI were failure of equipment to cross the lesion (68.8%), followed by failure of balloon expansion in 25%, and balloon rupture in 6.3% of the procedures. In most cases, one burr was enough for RA (87.5%), and two burrs were used in 12.5%. The largest burr size was 1.25 mm in 37.5% and 1.50 mm in 62.5%. The mean rotational speed for RA was 186,363 ± 12,863 RPM. There was no Rota wire uncrossing in our center and rotational atherectomy during CTO-PCI was successful in all 16 cases. Almost 75% of the access site in both groups were radical plus femoral. Dual/single radical and dual/single femoral constituted a low percentage in two groups. Technical (87.5% vs. 87.5%; *p* > 0.99) and procedural (87.5% vs. 85.9%; *p* > 0.99) success rates were similar between the RA and non-RA group. Other procedural metrics were similar between the two groups.

**Table 3:** Procedural characteristics.

**Variable**	**Non-RA** **(*n* = 313)**	**RA** **(*n* = 16)**	*P*-value^*^
Successful crossing technique			
Reverse CART/ Guidezilla^TM^ reverse CART, n (%)	186 (59.4)	12 (75)	0.29
Retrograde wire knuckle, *n* (%)	6 (1.9)	1 (6.3)	>0.99
Retrograde wire escalation, *n* (%)	85 (27.2)	3 (18.8)	0.57
Guidezilla^TM^ use, *n* (%)	144 (46.0)	12 (75)	0.04
IVUS use, *n* (%)	61 (19.5)	5 (31.3)	0.33
Channel type			
Epicardial collateral channel, *n* (%)	80 (25.6)	3 (18.8)	0.77
Septal collateral channel, *n* (%)	238 (76.0)	13 (81.3)	0.77
Number of stents implanted	2.50 ± 1.17	3.14 ± 0.86	0.09
Total stent length (mm)	90.69 ± 30.67	105.5 ± 30.59	0.71
Indication of RA			
Equipment failure-to-cross, *n* (%)	/	11 (68.8)	
Balloon failure-to-expand, *n* (%)	/	4 (25)	
Balloon rupture, *n* (%)	/	1 (6.3)	
Number of burrs used			
One, *n* (%)	/	14 (87.5)	
Two, *n* (%)	/	2 (12.5)	
Largest burr used (mm)			
1.25, *n* (%)	/	6 (37.5)	
1.50, *n* (%)	/	10 (62.5)	
Rotational speed, RPM	/	186,363 ± 12,863	
Rotational atherectomy success, *n* (%)	/	16 (100)	
Technical success, *n* (%)	274 (87.5)	14 (87.5)	>0.99
Procedural success, *n* (%)	269 (85.9)	14 (87.5)	>0.99
Access site			
Bilateral/unilateral radical (%)	22 (7.0)	1 (6.3)	>0.99
Radical+femoral (%)	232 (74.1)	12 (75)	>0.99
Bilateral/unilateral femoral (%)	59 (18.8)	3 (18.8)	>0.99
Procedure time, minute	169.3 ± 71.30	188.3 ± 69.52	0.81
Contrast volume (ml)	211.1 ± 61.58	195.87 ± 63.76	0.77

*Reverse CART, Reverse Controlled Anterograde Retrograde Tracking; IVUS, intravascular ultrasound; RPM, revolutions per minute;*

* *p < 0.05 is considered significant*.

### Procedural Complications and In-hospital Outcomes

Procedural complications and in-hospital outcomes were shown in Table 4. There was no difference concerning major procedural complications between groups (12.5% vs. 19.2%; *p* > 0.75). In the RA group, there was one target vessel perforation with tamponade case, and one branch vessel perforation case identified by angiography after RA procedure. Spring coils were implanted in the perforated cases. No in-hospital MACCEs was recorded in the RA group while there were eight MACCEs in the non-RA group (*p* > 0.99). 55 cases of vessel perforations/dissections were recorded in non-RA group including 18 target vessel perforations, 2 branch vessel perforations, 26 septal collateral vessel perforations and 15 epicardial collateral vessel perforations (one patient died from cardiac tamponade).

**Table 4:** Procedural complications and in-hospital outcome.

**Variable**	**Non-RA** **(*n* = 313)**	**RA** **(*n* = 16)**	***P*-value**
**Procedural complications**, ***n*** **(%)**	60 (19.2)	2(12.5)	0.75
Perforations/dissections, *n* (%)	55 (17.6)	2 (12.5)	>0.99
Target vessel, *n* (%)	18 (5.75)	1 (6.25)	>0.99
Branch vessel, *n* (%)	2 (0.6)	1 (6.25)	>0.99
Septal collateral vessel, *n* (%)	26 (8.3)	0 (0)	0.63
Epicardial collateral vessel, *n* (%)	15 (4.8)	0 (0)	>0.99
Covered stent implantation, *n* (%)	5 (1.6)	0 (0)	>0.99
Coiling, *n* (%)	9 (2.9)	2 (12.5)	0.09
Cardiac tamponade, *n* (%)	12 (3.8)	1 (6.25)	0.48
Stent thrombosis, *n* (%)	3 (1.0)	0 (0)	>0.99
Burr entrapment, *n* (%)	/	0 (0)	>0.99
Access complications, *n* (%)	2 (0.6)	0 (0)	>0.99
**In-hospital MACCE**, ***n*** **(%)**	8 (2.6)	0 (0)	>0.99
Death, *n* (%)	1 (0.3)	0 (0)	>0.99
Periprocedural MI, *n* (%)	0 (0)	0 (0)	>0.99
Target vessel recanalization, *n* (%)	0 (0)	0 (0)	>0.99
Stroke, *n* (%)	0 (0)	0 (0)	>0.99
Pericardiocentesis, *n* (%)	6 (1.9)	0 (0)	>0.99
Tamponade requiring surgery, *n* (%)	1 (0.3)	0 (0)	>0.99

*MI, myocardial infarction; MACCE, major adverse cardiac and cerebral events*.

### Clinical Outcomes During Follow-Up

The overall follow-up rate was 93%. The duration of follow-up of non-RA group was 26.47 ± 14.46 months while the follow-up period of the RA group was 30.22 ± 15.07 months. Table 5 demonstrated clinical outcomes during follow-up. No difference was found regarding MACCEs, reason of rehospitalization, all-cause mortality and so on. Details of the RA group patients were listed in Supplementary Tables 1–3.

**Table 5:** Clinical outcomes on follow-up.

	**Non-RA** **(*n* = 313)**	**RA** **(*n* = 16)**	***P-*value**
Major adverse cardiac and cerebral events, *n* (%)	35 (11.2)	1 (6.3)	>0.99
Cardiac death, *n* (%)	12 (3.8)	1 (6.3)	0.48
Target-vessel revascularization, *n* (%)	10 (3.2)	0 (0)	>0.99
Myocardial infarction, *n* (%)	6 (1.9)	0 (0)	>0.99
Stroke, *n* (%)	7 (2.2)	0 (0)	>0.99
Reason of rehospitalization, *n* (%)	55 (17.6)	3 (18.8)	>0.99
Heart failure, *n* (%)	12 (3.8)	2 (12.5)	0.14
Angina, *n* (%)	17 (5.4)	0 (0)	>0.99
Stroke, *n* (%) kidney failure, *n* (%)	9 (2.9) 2 (0.6)	0 (0) 0 (0)	>0.99 >0.99
Ventricular tachycardia, *n* (%)	1 (0.3)	0 (0)	>0.99
pacemaker implanting, *n* (%)	2 (0.6)	0 (0)	>0.99
Gastrointestinal Hemorrhage, *n* (%)	1 (0.3)	0 (0)	>0.99
Malignant tumor, *n* (%)	3(1.0)	0 (0)	>0.99
Surgery for other reasons, *n* (%)	3 (1.0)	0 (0)	>0.99
Routine health check, *n* (%)	5 (1.6)	1 (5.3)	0.26
All-cause mortality, *n* (%)	18 (5.8)	1 (6.3)	>0.99
Lost to follow-up, *n* (%)	23 (9.8)	0 (0)	0.62

*RA, Rotational atheretomy*.

In unadjusted analysis, there was no difference between the RA and non-RA groups in terms of survival (HR 1.58, 95% CI, 0.31–8.21, *p* = 0.65). Moreover, performing RA during retrograde CTO-PCI did not lead to an increase in the MACCE rate during follow-up (HR 0.99, 95% CI 0.35–2.79, *p* = *0*.99). Neither the overall rehospitalization rate (HR 1.27, 95% CI 0.44–3.67, *p* = 0.67) or the heart failure symptom induced (HR 0.44, 95% CI 0.02–8.28, *p* = 0.43), angina induced (*p* = 0.53) or arrythmia induced (*p* = 0.80) rehospitalization rate was significantly different between the two groups. Of the three patients hospitalized in the RA group, one was asymptomatic according to our telephonic follow-up, hence, the observed difference between the two groups might be biased. No significant differences were observed between the two groups in terms of the non-fatal MI rate (*p* = 0.54), target vessel recanalization rate (*p* = 0.46) or the stroke rate (*p* = 0.41).

**Figure 3:**
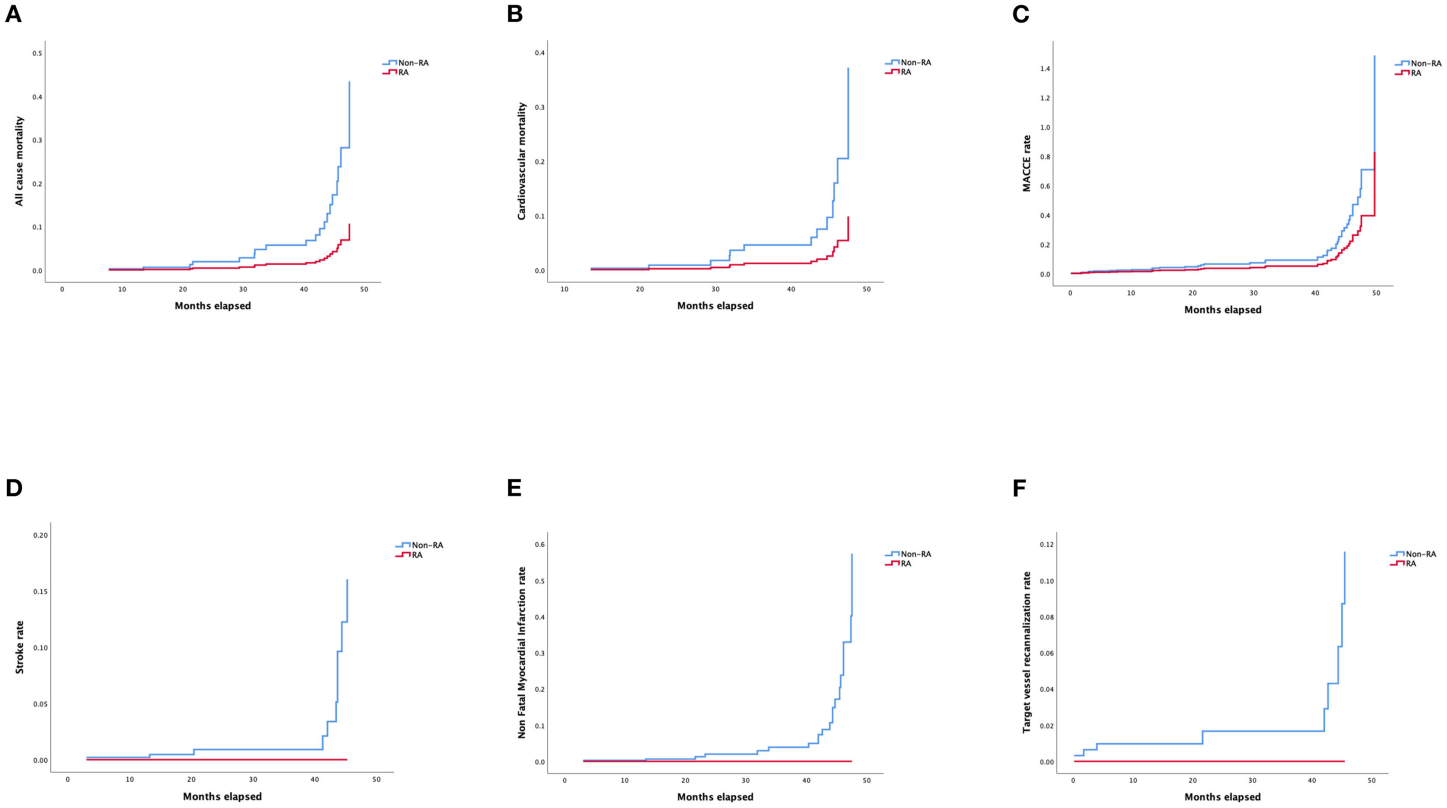
**(A)** Adjusted Kaplan–Meier curves of all-cause mortality according to Cox regression model. **(B)** Adjusted Kaplan–Meier curves of cardiovascular mortality according to Cox regression model. **(C)** Adjusted Kaplan–Meier curves of MACCE rate according to Cox regression model. **(D)** Adjusted Kaplan–Meier curves of stroke rate according to Cox regression model. **(E)** Adjusted Kaplan–Meier curves of non-fatal myocardial infarction rate according to Cox regression model. **(F)** Kaplan–Meier curves of target vessel recanalization rate according to Cox regression model.

**Figure 4:**
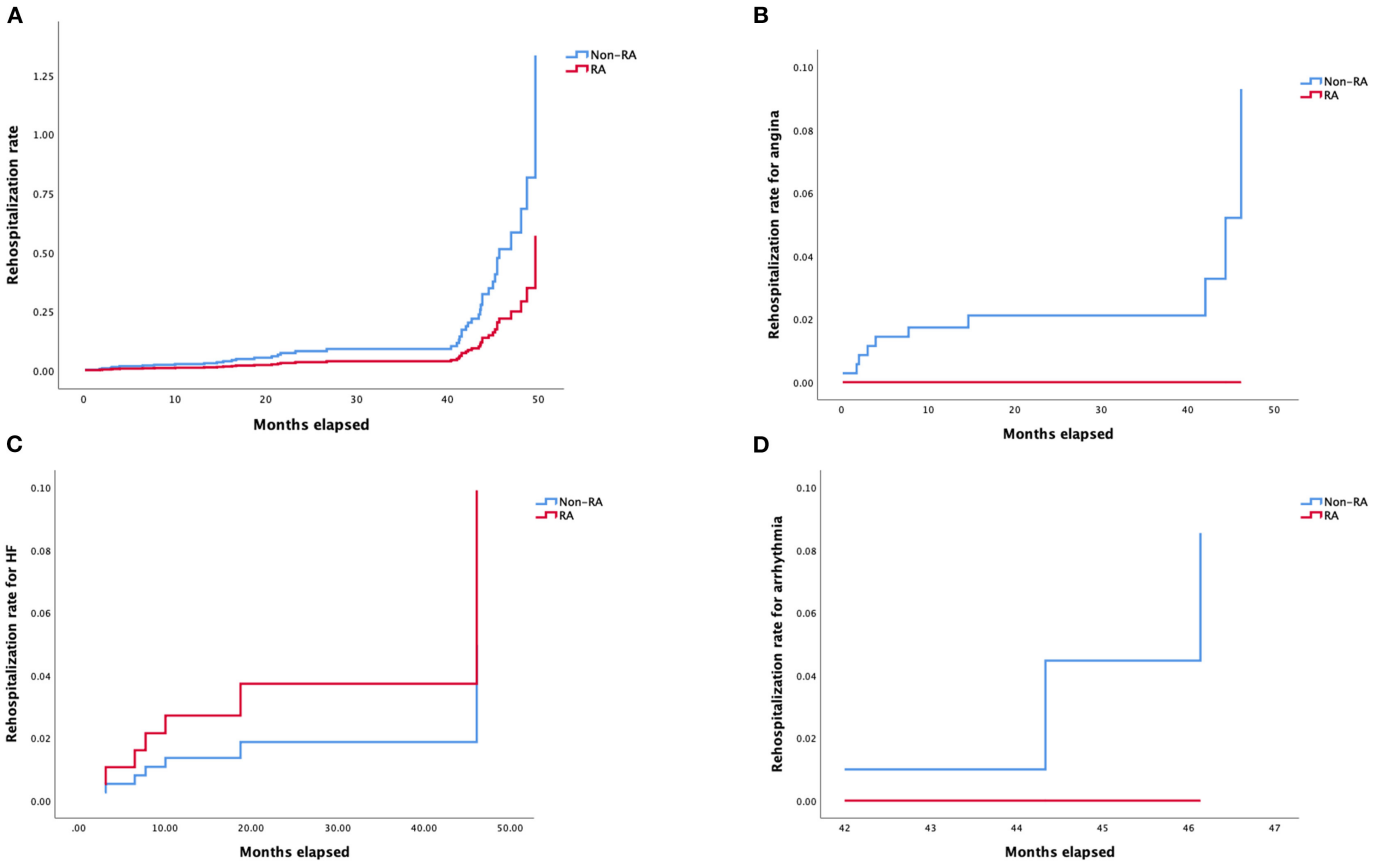
**(A)** Kaplan–Meier curves of rehospitalization rate according to Cox regression model. **(B)** Kaplan–Meier curves of rehospitalization rate for angina according to Cox regression model. **(C)** Kaplan–Meier curves of rehospitalization rate for heart failure according to Cox regression model. **(D)** Kaplan–Meier curves of rehospitalization rate for arrhythmia according to Cox regression model.

Details of multivariable stepwise Cox regression analysis adjusting for significant variables in univariable testing were listed in Supplementary Tables 4, 5. Adjusted Kaplan–Meier survival curves following retrograde CTO-PCI, with and without RA, was illustrated in Figure 3A. Hypertension (HR 3.65, 95% CI, 1.21–11.04, *p* = 0.02), prior MI (HR 3.40, 95% CI, 1.13–10.30, *p* = 0.03), multivessel and moderate/severe calcification (HR 2.98, 95% CI, 1.05–8.50, *p* = 0.04) were independent predictors of survival. Prior MI (HR 7.39, 95% CI, 1.79–30.48, *p* = 0.006), multivessel (HR 0.13, 95% CI, 0.02–0.78, *p* = 0.03) and moderate/severe calcification (HR 5.02, 95% CI, 1.18–21.31, *p* = 0.03) were independent predictors of the cardiovascular mortality rate during follow-up (Figure 3B). Hypertension (HR 3.51, 95% CI, 1.59–7.72, *p* = 0.002) and prior MI (HR 2.53, 95% CI, 1.24–5.17, *p* = 0.01) were independent predictors of the MACCE rate during follow-up (Figure 3C). Multivessel (HR 0.21, 95% CI, 0.05–0.84, *p* = 0.03) was the independent predictor of stroke rate during follow-up (Figure 3D). Moderate/severe calcification (HR 7.77, 95% CI, 0.90–67.21, *p* = 0.06) was the independent predictor of non-fatal myocardial infarction rate during follow-up (Figure 3E). Prior failed PCI (HR 4.92, 95% CI, 1.05–22.97, *p* = 0.04) was the independent predictor target vessel recanalization rate (Figure 3F). Figure 4 illustrated the Kaplan–Meier curves of adjusted overall rehospitalization rate (Figure 4A) and angina (Figure 4B), heart failure (Figure 4C) and arrhythmias (Figure 4D) caused rehospitalization rate. In the overall rehospitalization rate, moderate/severe calcification (HR 2.78, 95% CI, 1.33–5.82, *p* = 0.007) was an independent predictor.

## Discussion

To the best of our knowledge, this is the first study to compare the outcomes (in-hospital and long-term) of the use of RA in CTO-PCI using the retrograde approach. One of the main findings of our study was that the procedural success rate of retrograde CTO PCI was similar whether or not RA was performed. Additionally, we found that thought RA was used more often in patients with extreme vessel tortuosity and calcification, cases in both RA and non-RA groups performing retrograde CTO PCI were multivessel disease, multi-CTO lesions, and complex lesions (evidenced by J-CTO score and PROGRESS-CTO score) which can be regarded as CHIP (Complete Revascularization for High Risk Indicated Patients Session). We found similar rates of in-hospital complications as well as long-term survival, stroke, MI and MACCE rates with or without the use of RA during retrograde CTO-PCI despite similar rates of use of reverse-CART in the two groups. The rehospitalization rate was not higher when RA was used.

Balloon un-crossable or un-dilatable lesions account for 9 and 2% of CTO-PCI failure, respectively (20). Strategies such as the buddy wire technique, deep intubation of the guiding catheter, and the mother-and-child guide catheter techniques are frequently employed to facilitate device advancement during the procedure. RA can be very useful in this setting by debulking lesions with severe calcification to improve vascular compliance and device trafficability. A recent multicentral study suggested that excimer laser coronary atherectomy was effective in uncrossable CTO lesions (21). Although there is a lack of sufficient practice, we are optimistic about its prospects.

Recent randomized controlled trials have suggested that RA for complex calcified lesions was similar to that of plain old balloon angioplasty with regards to long-term clinical outcomes or reduction in lumen loss (4, 22, 23). Abdel-Wahab et al. have reported similar rates of immediate and 9-month lumen loss when modified balloons or RA were used in severely calcified coronary lesions (5). However, a recent *post hoc* analysis of the PREPARE-CALC (The Comparison of Strategies to PREPARE Severely CALCified Coronary Lesions) randomized trial found that RA had a higher procedural success rate compared with modified balloons in non-left anterior descending artery lesions (24). A retrospective review of 3540 patients in 21 centers (part of the PROGRESS-CTO registry) identified 116 patients in whom RA was performed and 3424 patients without RA, using both the antegrade and retrograde approaches to CTO-PCI (7). In this study, the technical and procedural success rates and MACCE rates were similar between the groups. RA was used in only 4.9% of patients undergoing retrograde CTO-PCI in our center, similar to the rate in the PROGRESS-CTO registry. This indicates that whilst RA is not a necessity in CTO-PCI, it nevertheless remains an effective and useful option in the occasional resistant lesion. A small study (*n* = 285) suggested a trend toward a lower 1-year MACCE rate when RA was used in resistant CTO lesions, but this advantage was lost after adjusting for confounding variables on multivariate Cox regression analysis (HR 1.25, 95% CI, 0.33–1.94, *p* = 0.242) (9). Similar to our study, a single-center study in Germany that enrolled CTO (*n* = 75) and non-CTO (*n* = 317) PCI patients who had RA prior to stent implantation found no differences in in-hospital MACCE rates despite the occurrence of significantly more dissections when RA was employed (8). In an in-hospital cohort of 129 patients undergoing CTO-PCI with RA reported a higher incidence of dissection in the retrograde arm compared with the antegrade approach (25).

In this study, the technical success rate of RA procedural was 100%, which we attribute, amongst other things, to the good support afforded by 7F guiding catheters and guide catheter-extensions (Guidezilla^TM^), as necessary, through unilateral radial and femoral artery or bilateral femoral artery vascular access sites. We analyzed the access site of the two groups and the result suggesting that in retrograde CTO-PCI surgery operators tended to apply right radial plus femoral artery. Comparing with bilateral radial artery and bilateral femoral artery, this method could provide a stronger support for catheters with less puncture complications and restriction after surgery (26). The right access is more feasible for the operators to stand a long time in the operation. For those lesions with severe tortuosity and calcification, the microcatheter often failed to cross in the antegrade approach (to facilitate wire exchange). In these cases, sophisticated operators would trace the track left by the stiff wire in the calcified lesion with Rotawire in the antegrade microcatheter and return to the distal true lumen. As the lesion was usually severely calcified, a track was usually left in the lesion without elastic recoil (19). During this procedure, retrograde angiography was performed once the Rotawire crossed the lesion to ensure that it was in the true lumen to reduce the risk of perforation (12). The smallest-sized burr (1.25 mm) was used at a speed of 160,000-180,000 RPM. In our study, reverse CART was used significantly more frequently in the RA group. As the lesions in the RA group were longer and more calcified, antegrade CTO wires were more prone to cross into the subadventitial space with inability to return to the true lumen (11). In this scenario, we switched over to the retrograde approach for recanalization, which likely accounted for the more frequent use of reverse CART in the RA group. Reverse CART allows the guide wire to travel through the subadventitial space and could lead to extension of the dissection plane and predisposition to perforation. This was confirmed by the findings of our study. However, we also found that the technical success, long term MACCE, and survival rates were similar, independent of the use of RA (27). Guidezilla^TM^ usage was higher in the RA group due to more severe calcification and tortuosity of the target vessels. Deep intubation of the Guidezilla^TM^ helped to direct the retrograde guide wire following CTO crossing (28). After successful retrograde wire externalization, the burr was delivered for RA. During the procedure, the Guidezilla^TM^ provided stability, maintained coaxiality with the Rotawire, and protected proximal branches during RA in distal lesions.

We found two cases regarded as procedural failure. Both two cases were due to vessel perforation and needed endovascular coiling to arrest the bleed. One of the two cases of perforation developed into pericardial tamponade requiring pericardial drainage. None of the two patients experienced adverse cardiovascular or cerebral events. Historically, a feared complication of RA has been enlargement of a subintimal dissection. We used reverse CART and wire knuckle techniques for retrograde CTO lesion crossing. This study found two cases of dissection in the RA group, but was not significantly higher than the rate recorded in the non-RA group. Research suggests that low-speed (140,000 RPM) RA did not result in a reduction in the slow flow phenomenon compared with high-speed (190,000 RPM) RA (29). But one successful case in the RA group reported cardiac death during rehospitalization. Overall, procedural success and MACCE rates were not different between the two groups in-hospital and during follow up.

Our study had some limitations. First, as a single-center retrospective study, the lack of randomization and potential for selection bias during the procedure might have affected the study outcomes. Second, the operations were performed by skillful operators in a large center and may not applicable to those small centers. Additionally, the number of patients who underwent RA during retrograde CTO-PCI was relatively small. Follow-up angiography was not performed for our patients. In this retrospective study, economic considerations in the past may lower the usage rate of IVUS, but our practice suggested that IVUS was a vital approach in the RA procedure for e.g., the selection of burr and RA guidewire, prevention of coronary perforation, evaluation of RA, and selection of the stent (30). IVUS was applied in complicating CTO lesions if possible. As a result, the true benefits of RA in CTO-PCI using the retrograde approach need to be further assessed by larger studies or dedicated randomized trials.

## Conclusion

In summary, our study demonstrates that the in-hospital outcomes and long-term follow up events were the same between RA and non-RA retrograde CTO-PCI patients. RA offered an option for skillful operators in difficult cases when the lesion was severely calcified in retrograde CTO-PCI.

## Data Availability Statement

The original contributions presented in the study are included in the article/Supplementary Material, further inquiries can be directed to the corresponding author.

## Ethics Statement

The studies involving human participants were reviewed and approved by the Research Ethics Committee of Guangdong Provincial People's Hospital and Guangdong Academy of Medical Sciences [No. GDREC2017196H(R1)]. The patients/participants provided their written informed consent to participate in this study.

## Author Contributions

JW designed the topic and revised the manuscript. BZ were involved in the conception and design of this study. ZZ and HL provided data and gave advice to the design of the research. KW and ZH gave advice to the reversion of the manuscript. A-SY revised the manuscript. JH analyzed the data and wrote the draft. All authors contributed to the article and approved the submitted version.

## Funding

This study was supported by the Science and Technology Planning Project of Guangdong Province, China (Grant No: 2016A020216022).

## Conflict of Interest

The authors declare that the research was conducted in the absence of any commercial or financial relationships that could be construed as a potential conflict of interest.

## Publisher's Note

All claims expressed in this article are solely those of the authors and do not necessarily represent those of their affiliated organizations, or those of the publisher, the editors and the reviewers. Any product that may be evaluated in this article, or claim that may be made by its manufacturer, is not guaranteed or endorsed by the publisher.

## References

[1] Galassi AR Sianos G Werner GS Escaned J Tomasello SD Boukhris M. Retrograde recanalization of chronic total occlusions in Europe: procedural, in-hospital, and long-term outcomes from the Multicenter ERCTO Registry. J Am Coll Cardiol. (2015) 65:2388–400. 10.1016/j.jacc.2015.03.566 26046732

[2] Levine GN Bates ER Blankenship JC Bailey SR Bittl JA Cercek B. 2011 ACCF/AHA/SCAI guideline for percutaneous coronary intervention. A report of the American college of cardiology foundation/American heart association task force on practice guidelines and the society for cardiovascular angiography and interventions. J Am Coll Cardiol. (2011) 58:e44–122. 10.1161/CIR.0b013e31823ba622 22070834

[3] Moussa I Di Mario C Moses J Reimers B Di Francesco L Martini G. Coronary stenting after rotational atherectomy in calcified and complex lesions. Angiographic and clinical follow-up results. Circulation. (1997) 96:128–36. 10.1161/01.CIR.96.1.128 9236427

[4] Abdel-Wahab M Richardt G Joachim Büttner H Toelg R Geist V Meinertz T. High-speed rotational atherectomy before paclitaxel-eluting stent implantation in complex calcified coronary lesions: the randomized ROTAXUS (Rotational Atherectomy Prior to Taxus Stent Treatment for Complex Native Coronary Artery Disease) trial. JACC Cardiovasc Interv. (2013) 6:10–9. 10.1016/j.jcin.2012.07.017 23266232

[5] Abdel-Wahab M Toelg R Byrne R Geist V El-Mawardy M Allali A. High-speed rotational atherectomy versus modified balloons prior to drug-eluting stent implantation in severely calcified coronary lesions. Circ Cardiovasc Interv. (2018) 11:e007415. 10.1161/CIRCINTERVENTIONS.118.007415 30354632

[6] Tajti P Karmpaliotis D Alaswad K Toma C Choi JW Jaffer FA. Prevalence, presentation and treatment of 'balloon undilatable' chronic total occlusions: insights from a multicenter US registry. Catheter Cardiovasc Interv. (2018) 91:657–66. 10.1002/ccd.27510 29359452 PMC5849516

[7] Xenogiannis I Karmpaliotis D Alaswad K Jaffer FA Yeh RW Patel M. Usefulness of atherectomy in chronic total occlusion interventions (from the PROGRESS-CTO Registry). Am J Cardiol. (2019) 123:1422–8. 10.1016/j.amjcard.2019.01.054 30798947

[8] Brinkmann C Eitan A Schwencke C Mathey D Schofer J. Rotational atherectomy in CTO lesions: too risky? Outcome of rotational atherectomy in CTO lesions compared to non-CTO lesions. EuroIntervention. (2018) 14:e1192–e8. 10.4244/EIJ-D-18-00393 30175961

[9] Huang WC Teng HI Chan WL Lu TM. Short-term and long-term clinical outcomes of rotational atherectomy in resistant chronic total occlusion. J Interv Cardiol. (2018) 31:458–64. 10.1111/joic.12489 29315803

[10] Christopoulos G Karmpaliotis D Alaswad K Yeh RW Jaffer FA Wyman RM. Application and outcomes of a hybrid approach to chronic total occlusion percutaneous coronary intervention in a contemporary multicenter US registry. Int J Cardiol. (2015) 198:222–8. 10.1016/j.ijcard.2015.06.093 26189193 PMC4554818

[11] Azzalini L Carlino M Brilakis ES Vo M Rinfret S Uretsky BS. Subadventitial techniques for chronic total occlusion percutaneous coronary intervention: the concept of vessel architecture. Catheterization Cardiovasc Interv. (2018) 91:725–34. 10.1002/ccd.27025 28303648

[12] Capretti G Carlino M Colombo A Azzalini L. Rotational atherectomy in the subadventitial space to allow safe and successful chronic total occlusion recanalization: pushing the limit further. Catheter Cardiovasc Interv. (2018) 91:47–52. 10.1002/ccd.27035 28417604

[13] Iannaccone M Colangelo S Colombo F Garbo R. Rotational atherectomy with the new RotaPro system over RG3 guidewire in subadventitial retrograde highly calcified CTO PCI. Catheter Cardiovasc Interv. (2020) 95:242–4. 10.1002/ccd.28438 31403252

[14] Morino Y Abe M Morimoto T Kimura T Hayashi Y Muramatsu T. Predicting successful guidewire crossing through chronic total occlusion of native coronary lesions within 30 minutes: the J-CTO (Multicenter CTO Registry in Japan) score as a difficulty grading and time assessment tool. JACC Cardiovasc Interv. (2011) 4:213–21. 10.1016/j.jcin.2010.09.024 21349461

[15] Werner GS Ferrari M Heinke S Kuethe F Surber R Richartz BM. Angiographic assessment of collateral connections in comparison with invasively determined collateral function in chronic coronary occlusions. Circulation. (2003) 107:1972–7. 10.1161/01.CIR.0000061953.72662.3A 12665484

[16] Christopoulos G Kandzari DE Yeh RW Jaffer FA Karmpaliotis D Wyman MR. Development and validation of a novel scoring system for predicting technical success of chronic total occlusion percutaneous coronary interventions: the PROGRESS CTO (Prospective Global Registry for the Study of Chronic Total Occlusion Intervention) score. JACC Cardiovasc Interv. (2016) 9:1–9. 10.1016/j.jcin.2015.09.022 26762904

[17] Alpert JS. The fourth edition of the universal definition of myocardial infarction. Am J Med. (2018) 131:1265–6. 10.1016/j.amjmed.2018.06.016 30012357

[18] Cutlip DE Windecker S Mehran R Boam A Cohen DJ van Es GA. Clinical end points in coronary stent trials: a case for standardized definitions. Circulation. (2007) 115:2344–51. 10.1161/CIRCULATIONAHA.106.685313 17470709

[19] Zhang B Wang F Tan JWC Liao H Chai W Yu H. The application of rotational atherectomy in PCI of coronary chronic total occlusions. ASEAN Heart J. (2016) 24:1. 10.7603/s40602-016-0001-8

[20] Stone GW Reifart NJ Moussa I Hoye A Cox DA Colombo A. Percutaneous recanalization of chronically occluded coronary arteries: a consensus document: part II. Circulation. (2005) 112:2530–7. 10.1161/CIRCULATIONAHA.105.583716 16230504

[21] Ojeda S Azzalini L Suárez de Lezo J Johal GS González R Barman N. Excimer laser coronary atherectomy for uncrossable coronary lesions A multicenter registry. Catheter Cardiovasc Interv. (2021) 98:1241–9. 10.1002/ccd.29392 33232583

[22] vom Dahl J Dietz U Haager P Silber S Niccoli L Buettner HJ. Rotational atherectomy does not reduce recurrent in-stent restenosis: results of the angioplasty versus rotational atherectomy for treatment of diffuse in-stent restenosis trial (ARTIST). Circulation. (2002) 105:583–8. 10.1161/hc0502.103347 11827923

[23] de Waha S Allali A Büttner HJ Toelg R Geist V Neumann FJ. Rotational atherectomy before paclitaxel-eluting stent implantation in complex calcified coronary lesions: two-year clinical outcome of the randomized ROTAXUS trial. Catheterization Cardiovasc Interv. (2016) 87:691–700. 10.1002/ccd.26290 26525804

[24] Rheude T Toelg R Byrne RA Allali A Wiebe J Sulimov DS. Outcomes of rotational atherectomy versus modified balloon angioplasty in severely calcified coronary lesions based on target lesion location: a post hoc analysis of the PREPARE-CALC randomised trial. EuroIntervention. (2020) 16:e322–e4. 10.4244/EIJ-D-19-00488 31566573

[25] Xu R Song X Chang S Qin Q Li C Fu M. Procedural and in-hospital outcomes of rotational atherectomy in retrograde coronary chronic total occlusion intervention. Angiology. (2021) 72:44–9. 10.1177/0003319720949312 32799665

[26] Tajti P Alaswad K Karmpaliotis D Jaffer FA Yeh RW Patel M. Procedural Outcomes of Percutaneous Coronary Interventions for Chronic Total Occlusions Via the Radial Approach: Insights From an International Chronic Total Occlusion Registry. JACC Cardiovasc Interv. (2019) 12:346–58. 10.1016/j.jcin.2018.11.019 30784639

[27] Azzalini L Dautov R Ojeda S Serra A Benincasa S Bellini B. Long-term outcomes of rotational atherectomy for the percutaneous treatment of chronic total occlusions. Catheter Cardiovasc Interv. (2017) 89:820–8. 10.1002/ccd.26829 28029214

[28] Huang Z Zhang B Chai W Ma D Liao H Zhong Z. Usefulness and safety of a novel modification of the retrograde approach for the long tortuous chronic total occlusion of coronary arteries. Int Heart J. (2017) 58:351–6. 10.1536/ihj.16-337 28539570

[29] Sakakura K Funayama H Taniguchi Y Tsurumaki Y Yamamoto K Matsumoto M. The incidence of slow flow after rotational atherectomy of calcified coronary arteries: a randomized study of low speed versus high speed. Catheter Cardiovasc Interv. (2017) 89:832–40. 10.1002/ccd.26698 27453426

[30] Sakakura K Yamamoto K Taniguchi Y Tsurumaki Y Momomura SI Fujita H. Intravascular ultrasound enhances the safety of rotational atherectomy. Cardiovasc Revasc Med. (2018) 19:286–91. 10.1016/j.carrev.2017.09.012 29113866

