# Supplementary material for: Safety and Feasibility of Rotational Atherectomy for Retrograde Recanalization of Chronically Occluded Coronary Arteries
Source: Front Cardiovasc Med. 2022 Jun 17;9:854757. doi: 10.3389/fcvm.2022.854757 (PMC9247204; doi:10.3389/fcvm.2022.854757)
Supplement: Supplementary file 1 [file Table_1.docx]

**Supplement table 1.** **Baseline Characteristics of the Included RA group Patients**

| Patient number | Gender | Age, years | Comorbidity | | CCS class | PCI history | Smoking | LVEF (%) | LVDd (mm) |
| --- | --- | --- | --- | --- | --- | --- | --- | --- | --- |
| 1 | Male | 61 | HT/DD | III | | N | Y | 67 | 47 |
| 2 | Male | 54 | DD/PMI | II | | Y | Y | 62 | 42 |
| 3 | Male | 63 | HT/DD/DM | II | | Y | Y | 60 | 47 |
| 5 | Male | 59 | HT/PMI | III | | Y | Y | 42 | 62 |
| 4 | Male | 74 | HT/DM/DD | III | | N | Y | 65 | 50 |
| 6 | Male | 53 | HT/DM | II | | Y | Y | 39 | 60 |
| 7 | Male | 71 | HT/DM | IV | | N | Y | 49 | 50 |
| 8 | Male | 72 | HT | III | | Y | Y | 66 | 48 |
| 9 | Male | 61 | DM | II | | Y | N | 36 | 59 |
| 10 | Male | 66 | HT/DM | II | | N | Y | 59 | 53 |
| 11 | Male | 51 | HT | III | | Y | Y | 60 | 54 |
| 12  13  14  15  16 | Male  Male  Male  Male  Male | 55  63  36  73  62 | HT  HT/DM  HT  HT/DM/PMI  HT/DD/PMI | IV  IV  IV  IV  III | | N  Y  Y  Y  N | Y  Y  Y  Y  Y | 51  50  54  51  28 | 56  44  62  53  55 |

Abbreviations: NA, not available; Y, yes; N, no; HT, hypertension; DM, diabetes mellitus; DD, dyslipidemia; PMI, Prior myocardial infarction; CCS, Canadian Cardiovascular Society; LVEF, left ventricular ejection fraction; LVDd, left ventricular end-diastolic diameter.
