# Supplementary material for: Safety and Feasibility of Rotational Atherectomy for Retrograde Recanalization of Chronically Occluded Coronary Arteries
Source: Front Cardiovasc Med. 2022 Jun 17;9:854757. doi: 10.3389/fcvm.2022.854757 (PMC9247204; doi:10.3389/fcvm.2022.854757)
Supplement: Supplementary file 2 [file Table_2.docx]

**Supplement table 2.** **Procedural Characteristics of the Included RA Group patients**

| Patient  number | Burr diameter  (mm) | Rotational  speed (RPM) | Target vessel | Dosage of contrast media(ml) | | Type of guide catheter | Access for RA | Multiple Vessel |  |
| --- | --- | --- | --- | --- | --- | --- | --- | --- | --- |
| 1 | 1.5 | 170000 | RCA | | 280 | 6F AL0.75 (R)  7F XB3.5 (F) | R | Y |  |
| 2 | 1.5 | 180000 | RCA | | 200 | 7F AL0.75 (R) 7F XB3.5 (F) | R | Y |  |
| 3 | 1.5 | NA | RCA | | 90 | 6F AL1.0 (R)  7F XB3.5 (F) | R | Y |  |
| 5 | 1.25 | NA | RCA | | 210 | 7F AL1.0 (R)  7F XB3.5 (F) | R | Y |  |
| 4 | 1.5 | NA | RCA | | 108 | 6F AL 0.75 (R)  7F XB3.5 (F) | R | Y |  |
| 6 | 1.25 | NA | RCA | | NA | 7F AL.75 (R) 7F XB3.5 (F) | R | Y |  |
| 7 | 1.5 | 180000 | RCA | | 300 | 7F AL 0.75 (R) 7F XB 3.5 (F) | R | Y |  |
| 8 | 1.5 | 200000 | RCA | | 180 | 7F AL0.75 (R) 7F XB3.5 (F) | R | Y |  |
| 9 | 1.25/1.25 | 190000 | LAD | | 260 | 7FXB3.5(F)  7FAL1.0(R) | F | Y |  |
| 10 | 1.5 | 160000 | LAD | | 200 | 7F EBU3.5 (R) | R | Y |  |
| 11 | 1.5 | NA | RCA | | 200 | 7F AL1.0 (F)  7F XB3.5 (F) | F | Y |  |
| 12 | 1.5 | 190000 | RCA | | 150 | 7F AL1.0 (F)  7F XB3.5 (F) | F | Y |  |
| 13 | 1.25 | 200000 | RCA | | 200 | 7F AL.75 (R)  7F XB3.5 (F) | R | Y |  |
| 14 | 1.25/1.5 | 190000 | RCA | | 100 | 7F AL1.0 (F) 7F XB3.5 (F) | F | Y |  |
| 15 | 1.25 | 190000 | LAD | | 200 | 7F XB3.5 (F) 7F AL1.0 (R) | F | Y |  |
| 16 | 1.25 | 200000 | LAD | | 260 | 7FXB3.0 (F)  7FAL0.75 (R) | F | Y |  |

Abbreviations: NA, not available; Y, yes; N, no; RA, rotational atherectomy; R, radial artery; F, femoral artery; RCA, right coronary artery; LAD, left anterior descending branch; RPM, revolutions per minute.
