# Supplementary material for: Safety and Feasibility of Rotational Atherectomy for Retrograde Recanalization of Chronically Occluded Coronary Arteries
Source: Front Cardiovasc Med. 2022 Jun 17;9:854757. doi: 10.3389/fcvm.2022.854757 (PMC9247204; doi:10.3389/fcvm.2022.854757)
Supplement: Supplementary file 3 [file Table_3.docx]

**Supplement ­­­table 3.** **Procedural and in-hospital details of retrograde RA patients**

| Patients | Target vessel | Discovery of perforation /dissection (IVUS or angiography) | Dissection before or after RA | Reason of rehospitalization | Accsess site | RA access site | PCI strategy | Cause of dissection |
| --- | --- | --- | --- | --- | --- | --- | --- | --- |
| 1  2  3  4  5  6  7  8  9  10  11  12  13  14  15  16 | RCA  RCA  RCA  RCA  RCA  RCA  RCA  RCA  LAD  LAD  RCA  RCA  RCA  RCA  LAD  LAD | N  N  N  N  N  N  N  N  Angiography  N  N  N  N  N  N  Angiography | N  N  N  N  N  N  N  N  After RA  N  N  N  N  N  N  After RA | N  N  N  Health check  Heart failure  N  N  N  N  N  N  Heart failure  N  N  N  N | R+F  R+F  R+F  R+F  R+F  R+F  R+F  R+F  R+F  R  F+F  R+F  R+F  F+F  R+F  R+F | R  R  R  R  R  R  R  R  F  R  F  F  R  F  F  F | Reverse-CART  Guidezilla-CART  Guidezilla-CART  Reverse-CART  Guidezilla-CART  Guidezilla-CART  Wire knuckle  Reverse-CART  Reverse-CART  Retro-wire escalation  Reverse-CART  Guidezilla-CART  Guidezilla-CART  Guidezilla-CART  Retro-wire escalation  Retro-wire escalation | **Patient 9**:The RA guide wire was located at the distal of the first diagonal branch. After RA, the diagonal branch was perforated and blocked by the spring coil. During the second RA procedure, RA guide wire was located at the distal true lumen of LAD and caused dissection of LAD because the distal of LAD was too small.  **Patient 16**:The RA guide wire is located at the distal ture lumen of LAD. The distal of LAD was too small. After RA, dissection and perforation occurred in LAD due to the small vessel and spring coil blockage was performed. |

Abbreviations: NA, not available; Y, yes; N, no; RCA, right coronary artery; LAD, left anterior descent branch; RA, rotational atherectomy; R, radical; F, femoral; IVUS, Intravascular ultrasound; CART, controlled anterograde retrograde tracking.
