# Supplementary material for: Safety and Feasibility of Rotational Atherectomy for Retrograde Recanalization of Chronically Occluded Coronary Arteries
Source: Front Cardiovasc Med. 2022 Jun 17;9:854757. doi: 10.3389/fcvm.2022.854757 (PMC9247204; doi:10.3389/fcvm.2022.854757)
Supplement: Supplementary file 4 [file Table_4.docx]

| Variables | Univariable | |  | Stepwise multivariable | |
| --- | --- | --- | --- | --- | --- |
|  | HR (95% CI) | p-value |  | HR (95% CI) | p-value |
| All-cause mortality rate |  |  |  |  |  |
| Hypertension  Dyslipidemia  Prior MI  Prior PCI | 3.56(1.19-10.70)  0.25(0.05-1.19)  3.21(1.26-8.19)  6.60(0.88-49.47) | 0.02  0.81  0.02  0.07 |  | 3.65(1.21-11.04)  0.38(0.08-1.89)  3.40(1.13-10.30)  7.32(0.96-55.89) | 0.02  0.24  0.03  0.06 |
| Multivessel  Moderate/severe calcification | 0.34(0.10-1.18)  2.89(1.14-7.34) | 0.09  0.03 |  | 0.29(0.07-1.27)  2.98(1.05-8.50) | 0.09  0.04 |
| Cardiovascular mortality rate |  |  |  |  |  |
| Hypertension  Dyslipidemia  Prior MI | 3.25(0.90-11.73)  0.13(0.02-1.21)  4.35(1.48-12.79) | 0.07  0.07  0.01 |  | 3.33(0.87-12.99)  0.18(0.02-1.80)  7.39(1.79-30.48) | 0.08  0.15  0.006 |
| Multivessel  Blunt stump  Moderate/severe calcification  Guidezilla^TM^ use | 0.20(0.05-0.74)  6.98(0.91-53.43)  5.19(1.56-17.32)  0.34(0.10-1.11) | 0.02  0.06  0.01  0.07 |  | 0.13(0.02-0.78)  4.09(0.46-36.43)  5.02(1.18-21.31)  0.27(0.06-1.10) | 0.03  0.21  0.03  0.06 |
| MACCE rate |  |  |  |  |  |
| Hypertension  Dyslipidemia  Prior MI | 3.29(1.51-7.16)  0.43(0.17-1.07)  2.01(1.00-4.03) | 0.003  0.07  0.05 |  | 3.51(1.59-7.72)  0.52(0.21-1.34)  2.53(1.24-5.17) | 0.002  0.176  0.01 |
| Moderate/severe calcification | 1.91(0.98-3.75) | 0.06 |  | 0.56(0.28-1.12) | 0.10 |
| Non-fatal myocardial infarction rate |  |  |  |  |  |
| Moderate/severe calcification | 7.77(0.90-67.21) | 0.06 |  | / | / |
| Stroke rate |  |  |  |  |  |
| Hypertension  Multivessel | 4.13(0.87-19.52)  0.19(0.05-0.77) | 0.07  0.02 |  | 3.36(0.71-16.03)  0.21(0.05-0.84) | 0.13  0.03 |
| Target vessel recanalization rate |  |  |  |  |  |
| Prior failed CTO PCI | 4.92(1.05-22.97) | 0.04 |  | / | / |
| Rehospitalization rate |  |  |  |  |  |
| Moderate/severe calcification | 2.78(1.33-5.82) | 0.007 |  | / | / |

**Supplement table 4.** **Predictors in univariable and stepwise multivariable Cox regression analysis**
