# Supplementary material for: Safety and Feasibility of Rotational Atherectomy for Retrograde Recanalization of Chronically Occluded Coronary Arteries
Source: Front Cardiovasc Med. 2022 Jun 17;9:854757. doi: 10.3389/fcvm.2022.854757 (PMC9247204; doi:10.3389/fcvm.2022.854757)
Supplement: Supplementary file 5 [file Table_5.docx]

| Variable | Unadjusted HR (95% CI) | p-value |  | Adjusted HR (95% CI) | p-value |
| --- | --- | --- | --- | --- | --- |
| All-cause mortality rate | 1.58(0.31-8.21) | 0.65 |  | 4.07(0.50-33.27) | 0.19 |
| Cardiovascular mortality rate | 1.21(0.18-7.89) | 0.86 |  | 3.77(0.43-32.72) | 0.23 |
| MACCE rate | 0.99(0.35-2.79) | 0.99 |  | 1.80(0.51-6.34) | 0.36 |
| Non-fatal myocardial infarction rate | NA | 0.54 |  | NA | 0.98 |
| Stroke rate | NA | 0.41 |  | NA | 0.98 |
| Target vessel recanalization rate  Rehospitalization rate | NA  1.27(0.44-3.67) | 0.46  0.67 |  | NA  2.34(0.70-7.82) | 0.99  0.17 |
| Rehospitalization rate for arrhythmias  Rehospitalization rate for angina  Rehospitalization rate for heart failure | NA  NA  0.44(0.02-8.28) | 0.80  0.53  0.43 |  | NA  NA  1.99(0.19-21.14) | 0.99  0.99  0.57 |

**Supplement table 5. Hazard ratio of clinical outcomes during follow-up**

Abbreviations: HR, Hazard Ratio; CI, Confidence Interval(s); NA, Not Available; MACCE, Major Adverse Cardiac and Cerebral Events.
